# Outcomes in culture positive and culture negative ascitic fluid infection in patients with viral cirrhosis: cohort study

**DOI:** 10.1186/1471-230X-8-59

**Published:** 2008-12-18

**Authors:** Lubna Kamani, Khalid Mumtaz, Umair S Ahmed, Ailia W Ali, Wasim Jafri

**Affiliations:** 1 Medicine Department, Aga Khan University Hospital, Karachi, Pakistan

## Abstract

**Background:**

Ascitic fluid infection (AFI) in cirrhotic patients has a high morbidity and mortality. It has two variants namely, spontaneous bacterial peritonitis (SBP) and culture negative neutrocytic ascites (CNNA). The aim of this study was to determine the outcome in cirrhotic patients with culture positive (SBP) and culture negative neutrocytic ascites.

**Methods:**

We analyzed 675 consecutive hepatitis B and/or C related cirrhosis patients with ascites admitted in our hospital from November 2005 to December 2007. Of these, 187 patients had AFI; clinical and laboratory parameters of these patients including causes of cirrhosis, Child Turcotte Pugh (CTP) score were recorded.

**Results:**

Out of 187 patients with AFI, 44 (23.5%) had SBP while 143 (76.4%) had CNNA. Hepatitis C virus (HCV) infection was the most common cause of cirrhosis in 139 (74.3%) patients. Patients with SBP had high CTP score as compared to CNNA (12.52 ± 1.45 vs. 11.44 ± 1.66); p < 0.001. Platelets count was low in patients with SBP (101 ± 53 × 10^9^/L) as compared to CNNA (132 ± 91 × 10^9^/L), p = 0.005. We found a high creatinine (mg/dl) (1.95 ± 1.0 vs. 1.44 ± 0.85), (p = 0.003) and high prothrombin time (PT) in seconds (24.8 ± 6.6 vs. 22.4 ± 7.2) (p = 0.04) in SBP as compared to CNNA. More patients with SBP (14/44; 31.8%) had blood culture positivity as compare to CNNA (14/143; 9.8%), p = 0.002. Escherichia. Coli was the commonest organism in blood culture in 15/28 (53.5%) patients. SBP group had a higher mortality (11/44; 25%) as compared to CNNA (12/143; 8.4%), p = 0.003. On multiple logistic regression analysis, creatinine >1.1 mg/dl and positive blood culture were the independent predictors of mortality in patients with SBP.

**Conclusion:**

Patients with SBP have a higher mortality than CNNA. Independent predictors of mortality in SBP are raised serum creatinine and a positive blood culture.

## Background

Ascitic fluid infections (AFI) are frequent and severe complication in cirrhotic patients and have a high morbidity and mortality. Two variants of AFI have been described in medical literature, 1) Spontaneous bacterial peritonitis (SBP) with polymorph nuclear (PMN) count >250/mm^3 ^and positive ascitic fluid culture without any evidence of external or intra-abdominal source of infection [1] and 2) Culture negative neutrocytic ascites (CNNA) with PMN > 250/mm^3 ^and a negative ascitic fluid culture [2].

The first description of SBP did not include those patients who had a negative ascitic fluid culture. The term CNNA was proposed in 1984 [2] and is considered a variant of SBP associated with lower mortality as compared to SBP [3]. Then it was decided that an ascitic fluid PMN count >250/mm^3 ^in the absence of evidence of abdominal infection is also a form of AFI even though the ascitic culture is negative [4].

SBP is a serious complication of end-stage liver disease, with a very high recurrence rate of up to 70% at 1 yr [5-7], and is seen in 8–27% of hospitalized patients with cirrhosis and ascites. Studies suggest that the in-hospital mortality in patients with SBP ranges from 20% to 40% [8,9].

There are few studies addressing the outcome in cirrhotic patients with AFI [3,4,9]. One of the studies found high creatinine and blood urea nitrogen (BUN) on admission to be associated with high mortality regardless of ascitic fluid culture positivity and type of organism [9].

There is a paucity of literature comparing the outcome in patients based on culture results, and factors predicting poor prognosis in cirrhotic patients with ascitic fluid infection. The available literature has included very limited number of patients with the two variants AFI and shows low mortality in CNNA as compared to SBP [3]. Moreover, majority of these studies are reported in patients with alcoholic cirrhosis [4].

We studied the outcome in the two variants of AFI in a large cohort of patients with viral causes of cirrhosis including hepatitis B and/or hepatitis C in a tertiary care University Hospital setting.

## Methods

We analyzed 675 consecutive patients with viral causes of cirrhosis along with ascites admitted at The Aga Khan University Hospital from November 2005 to December 2007. The medical records of patients admitted with symptoms and signs of ascitic fluid infection such as fever, abdominal pain and those with asymptomatic infection were retrieved. The data of their age, gender, clinical features, complications such as hepatic encephalopathy, upper GI bleed, hepatocellular carcinoma, etc was collected along with laboratory data and the Child Turcotte Pugh (CTP) score was calculated for all patients. All patients had liver cirrhosis diagnosed on clinical, hematological and biochemical laboratory parameters along with ultrasonological findings. 187 patients with ascitic fluid infections met the criteria in the form of either SBP or CNNA and they were included in the study. Only patients who had first episode of either SBP or CNNA were included in this study. Patients with secondary bacterial peritonitis, non-cirrhotics causes of ascites such as tuberculosis or malignancy were excluded. Those with cirrhosis and ascites who received systemic antibiotics within 30 days for any other infection (s) were also excluded from the study. Patients with cirrhosis due to causes other than hepatitis B, C or D viral infections were also excluded.

Diagnostic paracentesis was performed on the bed side by sterile method with a 21 G needle attached to 20 cc syringe, and then collected into Ethylene Diamine Tetra Acetate (EDTA) tube and analyzed within 3 hours of extraction. Ascitic fluid was then centrifuged in laboratory for 3 minutes for detailed report including total proteins, total and differential leukocyte count. Smear was performed and stained with Geimsa. At the same time 10 cc of ascitic fluid (5 cc in each bottle) was inoculated in aerobic and anaerobic blood culture bottles (Bactec 9240, Becton Dickinson, Ireland) containing Trypticase foy broth and processed according to manufacture's instructions and were observed for 1 month.

Similarly, blood cultures were also drawn in aerobic and anaerobic blood culture bottles, before starting antibiotics. All patients were treated with ceftriaxone 2 grams once daily and it was changed to appropriate antibiotic (s) based on less than 50% improvement in PMN count in the repeat ascitic fluid detailed report after 48 hours or culture sensitivity results of initial ascitic fluid. Recovery from the AFI was assessed on the basis of decrease in ascitic fluid PMN count to < 250/mm^3^.

This study is approved by the Ethics Review Committee (ERC) of Aga Khan University Hospital.

### Statistical Analysis

Statistical analysis was performed using the Statistical package for social science SPSS (Release 15.0, standard version, copyright^© ^SPSS; 1989–02). A descriptive analysis was performed for clinical features and results are presented as mean ± standard deviation for quantitative variables and numbers (percentages) for qualitative variables. χ^2 ^test and Fisher's exact test were used for categorical variables, while the independent sample t-test was applied for numerical variables. We used a one-way analysis of variance (ANOVA) for determining any statistical difference amongst the subgroups. Variables found to be statistically significant in the univariate analysis (p ≤ 0.25) were included in a multivariate stepwise logistic regression model. The model was constructed to identify independent predictors of mortality in patients with SBP and to obtain the odds ratio.

All p-values were two sided and considered as statistically significant if < 0.05.

## Results

**Figure 1:**
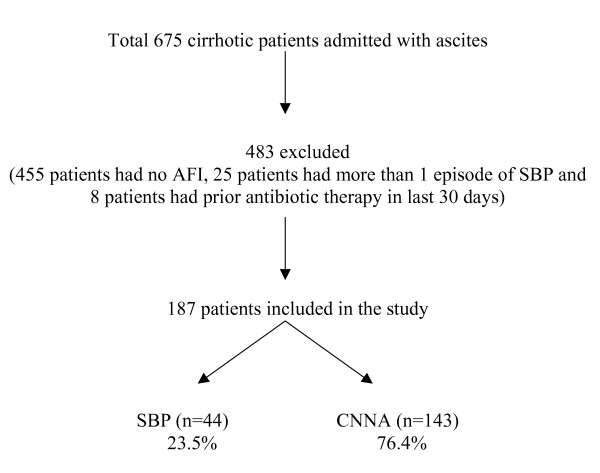
**Showing the distribution of patients with Cirrhosis and ascites**.

A total of 675 patients were admitted with ascites due to cirrhosis during the study period. Of these 187 (27.7%) patients with AFI were included for analysis. There were 44 (23.5%) patients who had SBP while 143 (76.4%) had CNNA (Figure 1).

The demographics and clinical data of patients with SBP and CNNA patients are shown in Table 1. The two groups of AFI patients, i.e., SBP and CNNA were matched for age, gender, clinical features, and complications such as upper GI bleed and hepatoma. The patients with SBP had statistically significant incidence of hepatic encephalopathy 36 (81.8%) vs. 83 (58%), p-value = 0.004; and had advanced CTP score 12.52 ± 1.45 at the time of admission as compared to CNNA group, 11.44 ± 1.66, p-value = <0.001.

**Table 1:** Demographics and clinical variables among patients with SBP and CNNA

**Variables**	**SBP (n = 44); ±/(%)**	**CNNA (n = 143); ±/(%)**	**p- value**
Age (years)	52.18 ± 10.6	53.49 ± 12.6	0.49
Gender			
Male	23(52.3)	76 (53.1)	
Female	21(47.7)	67 (46.9)	0.9
			
Fever	38(86.4)	125 (87.4)	0.8
Abdominal Pain	27(61.4)	80(55.9)	0.6
Encephalopathy	36 (81.8)	83(58)	0.004
GI bleed	5 (11.36)	14 (9.7)	0.10
CTP Score	12.52 ± 1.45	11.44 ± 1.66	<0.001
Hepatoma	7 (15.9)	39 (27.3)	0.12

Majority of hematological and biochemical laboratory tests including hemoglobin, white blood cells (WBC) counts, and liver function tests were similar in the two groups. There were significantly low levels of platelets in SBP group (101 ± 53 × 10^9^/L) as compared to CNNA (132 ± 91 × 10^9^/L), p-value = 0.005; similarly, SBP group had a high creatinine level (1.95 ± 1.0 mg/dl) as compared to CNNA (1.44 ± 0.85 mg/dl), p-value = 0.003. Ascitic fluid WBC counts were not different in the two groups, while blood cultures were significantly positive in patients with SBP. Table 2.

**Table 2:** Comparison of laboratory data of patents with SBP and CNNA

**Variables**	**SBP ± SD (n = 44)**	**CNNA ± SD (n = 143)**	**p- value**
Hemoglobin (g/dl)	10.43 ± 1.5	10.21 ± 1.9	0.44
WBC (10^9^/L)	10.9 ± 7.3	10.4 ± 5.6	0.69
Platelets (10^9^/L)	101 ± 53	132 ± 91	0.005
Creatinine (mg/dl)	1.95 ± 1	1.44 ± 0.85	0.003
Total Bilirubin (mg/dl)	7.82 ± 6.1	6.68 ± 6.8	0.30
SGPT (IU/L)	49.29 ± 41.4	60.58 ± 58.3	0.18
Prothrombin Time(s)	24.8 ± 6.6	22.4 ± 7.2	0.04
INR	2.05 ± 0.55	2.05 ± 2.5	0.99
Serum albumin (g/dl)	1.99 ± 0.37	2.04 ± 0.4	0.47
Ascitic TLC (cells/mm^3^)	4774 ± 3642.45	4384 ± 3638.71	0.70
Ascitic PMN (cells/mm^3^)	4055 ± 2860	3875 ± 2245	0.59
Blood culture positive	14 (31.8%)	14 (9.8%)	0.002

Hepatitis C virus (HCV) infection was the most common underlying cause of cirrhosis in 139 (74.3%) followed by hepatitis B virus (HBV) infection in 43 (22.9%); out of these 43 patients 12 (6.4%) had concomitant hepatitis D virus infection. Moreover, 5 (2.6%) patients had concomitant HBV and HCV infection causing cirrhosis.

Out of 187 patients with AFI, 44 (23.5%) had culture positive ascitic fluid cultures i.e., SBP; of these 32 (72.7%) patients had gram negative bacterial infection, while rest of 12 (27.2%) had gram positive infection. Escherichia. Coli (E. Coli) being the most common organism isolated from 27 (61.3%) patients. Table 3 shows the other organisms isolated from ascitic fluid cultures.

**Table 3:** Distribution of different organisms in patients with SBP (n = 44)

**Organisms cultured**	**n (%)**
E. Coli	27 (61.3)
Streptococcus Pneumoniae	5 (11.3)
Pseudomonas species	4 (9)
Staphylococcus species	3 (6.8)
Enterococcus species	3 (6.8)
Bacillus species	1 (2.2)
Group D Streptococcus	1 (2.2)

Overall 28/187 (14.9%) patients had positive blood culture results. The yield of culture was higher in SBP group (14/44; 31.8%) as compared to CNNA group (14/143; 9.8%), p-value = 0.002. There were 17 (60.7%) gram negative and 11 (39.2%) gram positive bacterial infection. Among the gram-negative organisms, E. coli was the most common isolated from blood culture in 15/28 (53.5%) patients.

All patients were started on intravenous ceftriaxone 2 grams once daily, after drawing ascitic and blood cultures. Intravenous salt poor albumin (25%), 100 ml twice a day for 5 days was given to all patients with a creatinine level of >1.5 mg/dl as per the protocol of our Gastroenterology Section. Out of 44 patients with SBP 13 were resistant to first line antibiotic; 7/32 (22%) were resistant in gram negative group while 6/12 (50%) in gram positive infection group. Gram negative resistant organisms were sensitive to other beta-lactam antibiotics; five of these patients were treated with piperacillin tazobactam and 2 received imepenam (carbepenam group) as per treating physician's decision. Similarly, vancomycin was given in 6 patients in whom ascitic culture was positive for Staphylococcus and Enterococcus species. Out of these 13 patients with resistant organism 8 died due to delay in start of appropriate antibiotics. There was 11/44 (25%) in-hospital deaths in SBP group of patients as compared to 12/143 (8.4%) in CNNA group, p-value = 0.003; the causes of death in SBP group were AFI and sepsis in 8 patients and PSE, hematemesis and seizures in 1 patient each. Whereas in CNNA 6 patients died of AFI and sepsis and 2 patients each in PSE, seizures and pneumonia

**Figure 2:**
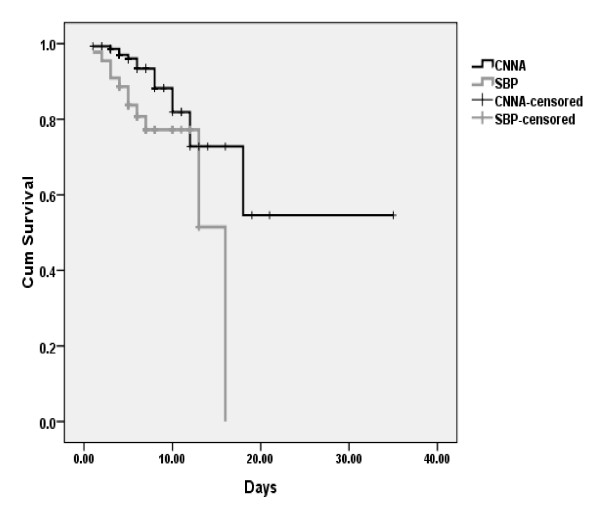
**Survival of patients and culture positive (SBP) and culture negative (CNNA)**.

Survival curve of patients with SBP and CNNA group of ascitic fluid infection is shown in Figure 2.

Predictors of mortality in patients with SBP variant of ascitic fluid infection, on univariate analysis were, presence of PSE, raised creatinine, low platelets counts, increased PT. Furthermore, blood culture positive results were also significant on univariate analysis for mortality in patients with SBP. We analyzed all these factors along with those of a p-value of <0.25 for finding the independent predictors of mortality by logistic regression technique. After multiple logistic regression analysis we found that a creatinine of >1.1 mg/dl and positive blood culture were the independent predictors of mortality in patients with SBP. Table 4

**Table 4:** Univariate and Multiple logistic regression analysis of predictors of mortality in SBP.

**Univariate Analysis:**			
Characteristics	Odd Ratio	95% CI	p

PSE	3.25	1.41 –7.49	0.006
Cr (>1.1 mg/dl)	3.65	1.29 – 10.29	0.01
Platelets (<91 × 109/L)	3.35	1.65 – 6.79	0.001
PT (>24 Secs)	2.18	0.92 – 5.13	0.005
Blood culture positivity	4.30	1.85 – 9.96	0.001

**Multiple logistic regression analysis:**			

Cr (>1.1 mg/dl)	5.78	1.23 – 27.1	0.02
Blood culture positivity	7.8	2.15 – 28.2	0.002

## Discussion

This is one of the largest reports of hepatitis B and C virus related cirrhotic patients with ascitic fluid infection. The incidence of AFI, including SBP and CNNA was found to be around 28%. We found that mortality is higher in patients with SBP 11/44 (25%) as compared to CNNA 12/143 (8.4%) in our series. We also found that the independent predictors of mortality in SBP were raised creatinine (> 1.1 mg/dl) along with blood culture positivity.

SBP and CNNA are both considered serious complications of advanced liver cirrhosis [10]. Reported incidence of AFI infection in cirrhotics ranges from 8–30% and mean in- hospital mortality is reported to reach 78% [1]. Incidence of AFI in our series was 28%, which is similar to reported literature.

Runyon and Hoefs in 1984 [2] studied 32 patients with a positive ascitic fluid culture and 17 patients with a negative ascitic fluid culture, with their mortalities being 70% and 50% respectively. There was however, no statistical difference noted. In another study, Pelletier et al in 1990 studied 15 patients with CNNA and 38 patients with SBP and showed the mortality to be considerably higher in patients with SBP [11]. In our study mortality rates were noted to be higher in patients with SBP (25%) than in CNNA (8.4%), possibly suggesting that CNNA is a less severe variant of SBP, despite the fact that both groups received similar intravenous third generation antibiotic and albumin. Different theories have been suggested for ascitic fluid infection severity. Impaired activity of the reticulo-endothelial system, decreased serum and ascitic complement levels, and a low ascitic protein level are some of the mechanisms responsible for entrance of enteral organisms into the ascitic fluid of patients with SBP [1]. Another established theory is that portal hypertension increases bacterial translocation to the lymphatic system and portal vein. The potential mechanisms responsible for this action are bacterial overgrowth due to impaired gastrointestinal transit, impaired host defense or morphological and functional damage to the bowel mucosa [12]. Lower mortality rates in patients with CNNA could be explained by a better hepatic function in terms of better CTP in patients with CNNA. We know that CTP score reflects the severity of portal hypertension and this could contribute to more bacterial translocation in patients with SBP.

In our report the rate of ascitic fluid bacterial growth was less (23%) as compared to other reports, where it ranges from 50 to 71% [3,11]. Use of blood culture bottles and the volume of the blood sample drawn affect the yield of culture. Sending a syringe or tube of fluid to the laboratory for culture dramatically decreases the sensitivity of the results since SBP is a low-colony-count monomicrobial infection similar to bacteremia [13]. Thus, culturing ascitic fluid as if it were blood (with bedside inoculation of ascitic fluid into blood culture bottles) has been shown to increase the culture-positivity of the ascitic fluid of patients with an ascitic fluid PMN count >250 cells/mm3 (in the absence of prior antibiotic treatment, pancreatitis, tuberculous peritonitis, or malignancy-related ascites) from about 50% to approximately 80% [13]^. ^We were unable to find any reason for this low yield accept that the amount of ascitic fluid inoculated was 5 cc in each bottle as compared to few studies where 10 cc was inoculated [4,11].

E. coli was the most common organism found in two thirds of patients with SBP, monomicrobial gram-negative organism was recovered in 32 (72.7%) patients and E. coli representing more than two thirds, which is similar to other studies [3] (Table 3). In our patients with SBP, blood culture yield was higher as compared to CNNA and it is comparable to other studies [4,11]. Interestingly, we found that, the death in patients resistant to first line antibiotic was high emphasizing on the need for an early detection method of organism for appropriate antibiotics according to sensitivity. Currently with an improvement in culture technique, time required for bacterial growth and its detection is usually more than 24 hours [14]. Considering the prognostic significance of early appropriate antibiotic treatment in patients with SBP [15], it would be pertinent to find a method to shorten the time for bacterial identification in clinical practice such as BacT/ALERT and bacterial DNA by PCR. BacT/ALERT is an automated microbial detection system, which is proven to be faster than conventional blood culture bottles in patients with bacteremia [16]. An earlier report of sensitivity of bacteria in ascitic fluid can be helpful in starting appropriate antibiotics, which can decrease mortality. In our study, raised serum creatinine and a positive blood culture were found to be the independent predictors of prognosis in SBP patients. Similarly Follo A et al reported renal impairment to be the most sensitive predicator of in hospital mortality in ascitic fluid infection [17].

Over all 23/187 (12.2%) patients with AFI died in our report; out of this majority, belonged to SBP group. The possible explanation for worse prognosis in SBP group could be advanced liver cirrhosis in this group as evidenced by CTP score and more renal impairment (Table 1 and 2). There is literature to support that renal impairment is a well-known prognostic factor of poor survival in patients with SBP [17]. Several authors suggested that CNNA and SBP are two variants of same disease with similar outcomes [4,18]. Our results together with report of Amri et al [3] and Pelletier at al [11] suggest SBP to be more severe variant with poor prognosis than CNNA. Early diagnosis of SBP along with prompt initiation of appropriate antibiotic therapy can be helpful in overall patient's survival.

## Conclusion

In conclusion ascitic fluid infection is a serious and life threatening complication of liver cirrhosis. SBP is associated with a higher mortality than CNNA. Patients with raised serum creatinine levels and positive blood cultures have poor prognosis, and they should be treated with greater vigilance. In the new era advanced culture methods with high and early yield can facilitate a prompt and accurate initiation of antibiotic therapy.

## Competing interests

The authors declare that they have no competing interests.

## Authors' contributions

LK and KM conceived the idea and drafted the protocol. LK supervised the data collection and statistical analysis; she wrote the first draft and revised manuscript under the supervision of KM. KM revised and advised for modifications in the statistical analysis and manuscript. USA and AWA were mainly involved in data collection and entered into SPSS software; they also contributed in the first draft of manuscript. WJ contributed in conception of study and participated in design and coordination. All authors read and approved the final manuscript.

## Pre-publication history

The pre-publication history for this paper can be accessed here:



## References

[B1] Hoefs JC, Runyon BA (1985). Spontaneous bacterial peritonitis. Dis Mon.

[B2] Runyon BA, Hoefs JC (1984). Culture-negative neutrocytic ascites: A variant of spontaneous bacterial peritonitis. Hepatology.

[B3] Saleh MA, Abdulkadir RA, Ibrahim AM (1994). Spontaneous bacterial peritonitis and culture negative neutrocytic ascites in patients with non-alcoholic liver cirrhosis. J Gastroenterol Hepatol.

[B4] Terg R, Levi D, Lopez P (1992). Analysis of clinical course and prognosis of culture positive spontaneous bacterial peritonitis and neutrocytic ascites. Evidence of the same disease. Dig Dis Sci.

[B5] Runyon BA (1998). Management of adult patients with ascites caused by cirrhosis. Hepatology.

[B6] Navasa M, Rodes J (1997). Management of ascites in the patient with portal hypertension with emphasis on spontaneous bacterial peritonitis. Semin Gastrointest Dis.

[B7] Guarner C, Sorian G (1997). Spontaneous bacterial peritonitis. Semin Liver Dis.

[B8] Sort P, Navasa M, Arroyo V (1999). Effect of intravenous albumin on renal impairment and mortality in patients with cirrhosis and spontaneous bacterial peritonitis. N Engl J Med.

[B9] Toledo C, Salmeron JM, Rimola A (1993). Spontaneous bacterial peritonitis in cirrhosis: Predictive factors of infection resolution and survival in patients treated with cefotaxime. Hepatology.

[B10] Runyon BA (1988). Spontaneous bacterial peritonitis: an explosion of information. Hepatology.

[B11] Pelletier G, Salmon D, Ink O (1990). Culture-negative neutrocytic ascites: A less severe variant of spontaneous bacterial peritonitis. J Hepatol.

[B12] Sorell WT, Quigley EM, Jin G, Johnson TJ, Rikkers LF (1993). Bacterial translocation in the portal-hypertensive rat: studies in basal conditions and on exposure to hemorrhagic shock. Gastroenterology.

[B13] Runyon BA, Canawati HN, Akriviadis EA (1988). Optimization of ascitic fluid culture technique. Gastroenterology.

[B14] Runyon BA, Hoefs JC (1984). Ascitic fluid analyses in the differentiation of spontaneous bacterial peritonitis from gastrointestinal tract perforation into ascitic fluid. Hepatology.

[B15] Such J, Runyon BA (1998). Spontaneous bacterial peritonitis. Clin Infect Dis.

[B16] Rohner P, Pepey B, Auckenthaler R (1995). Comparison of BacT/ALERT with signal blood culture system. J Clin Microbiol.

[B17] Follo A, Llovet JM, Navasa M (1994). Renal impairment after spontaneous bacterial peritonitis in cirrhosis: incidence, clinical course, predictive factors and prognosis. Hepatology.

[B18] Wyke Rj (1987). Problems of bacterial infection in patients with liver disease. Gut.

